# Acknowledgement of manuscript reviewers 2014

**DOI:** 10.1186/s12937-015-0003-6

**Published:** 2015-03-11

**Authors:** Nehme Gabriel

**Affiliations:** Nutrition Journal, Florida, USA

## Abstract

The editor of *Nutrition Journal* would like to thank all our reviewers who have contributed to the journal from Volume 13 (2014).

**Jean Abed**

United States of America

**Arianna Aceti**

Italy

**Dirk Aerenhouts**

Belgium

**Haleama Al Sabbah**

United Arab Emirates

**Ute Alexy**

Germany

**Bettaieb Ali**

France

**Gjumrakch Aliev**

United States of America

**Alessandro Antonelli**

Italy

**Jose Antonio**

United States of America

**Georgios Antonoglou**

Finland

**Hidekazu Arai**

Japan

**Trevor Archer**

Sweden

**Shervin Assari**

United States of America

**Hans-Jörg Assion**

Germany

**Deniz Atalayer**

United States of America

**Dagfinn Aune**

United Kingdom

**Fazli Awan**

Pakistan

**Max Oscar Bachmann**

United Kingdom

**Inkyung Baik**

Korea, South

**Pat Baird**

United States of America

**William Baker**

United States of America

**Riccardo Barbagallo**

Italy

**Neal Barnard**

United States of America

**Joshua Barzilay**

United States of America

**Nadia Bastide**

France

**Jamie Baum**

United States of America

**Jacquie Bay**

New Zealand

**Adrianne Bendich**

United States of America

**Judit Bene**

Hungary

**Jean-Jacques Benoliel**

France

**Rolf Kristian Berge**

Norway

**Joline Beulens**

Netherlands

**Ilana Bezerra**

Brazil

**Diane Birt**

United States of America

**Simona Bo**

Italy

**Pawel Bogdanski**

Poland

**Celine Bonnet**

France

**Ingvar Bosaeus**

Sweden

**Rachel Brown**

New Zealand

**Thiago Bruder-Nascimento**

Brazil

**Richard Bruno**

United States of America

**My Bui**

United States of America

**Victoria Bunik**

Russian Federation

**Mona Calvo**

United States of America

**María Alicia Camina Martín**

Spain

**T Colin Campbell**

United States of America

**Monica H. Carlsen**

Norway

**Maria Annunziata Carluccio**

Italy

**Timothy Carr**

United States of America

**Susana Casal**

Portugal

**Dick Chan**

Australia

**Ronna Chan**

United States of America

**Sybil Charriere**

France

**Benoit Chassaing**

United States of America

**Visith Chavasit**

Thailand

**Wayman Cheatham**

United States of America

**Guo-Chong Chen**

China

**Oliver Chen**

United States of America

**Yu-Han Chiu**

United States of America

**Kai-Chow Choi**

Hong Kong

**Jason Cholewa**

United States of America

**Efterpi Christaki**

Greece

**Ock K. Chun**

United States of America

**Gaudichon Claire**

France

**Miriam Clegg**

United Kingdom

**Roger Clemens**

United States of America

**Raquel Coelho**

Brazil

**Scott Collier**

United States of America

**Clare Collins**

Australia

**Emilie Combet**

United Kingdom

**Kevin Comerford**

United States of America

**Helen Coulthard**

United Kingdom

**Maria Covas**

Spain

**Daniel Crabtree**

United Kingdom

**Leslie Cunningham-Sabo**

United States of America

**Justyna Czech-Kowalska**

Poland

**Riccardo Dalle Grave**

Italy

**Dominique Dardevet**

France

**Undurti Das**

United States of America

**Srinivasa Raju Datla**

United States of America

**Luc Dauchet**

France

**Brenda Davy**

United States of America

**Willem De Keyzer**

Belgium

**Andrea De Silva**

Australia

**Rafael Deminice**

Brazil

**Elizabeth Dennis**

United States of America

**Peter Deriemaeker**

Belgium

**Laura Deroma**

Italy

**Jonathan Devries**

United States of America

**Ram Dickman**

Israel

**James Dinicolantonio**

United States of America

**Gustav Dobos**

Germany

**Lorenzo M Donini**

Italy

**Fiona Doolan-Noble**

New Zealand

**Vicky Drapeau**

Canada

**Pin-Der Duh**

Taiwan

**Leticia Elizondo-Montemayor**

Mexico

**Sean Fair**

Ireland

**Frida Fåk**

Sweden

**Ioannis Fatouros**

Greece

**Sally Ferguson**

Australia

**Brigite Ferreira**

Portugal

**Robyn Flipse**

United States of America

**Ewa Florek**

Poland

**Maria João Fonseca**

Portugal

**Maelán Fontes Villalba**

Spain

**Adrian Franke**

United States of America

**Xueyan Fu**

United States of America

**Ellen Fung**

United States of America

**Ujjawal Gandhi**

United States of America

**Vijay Ganji**

United States of America

**Parveen Garg**

United States of America

**Genevieve Gariepy**

Canada

**Glen Gibson**

United Kingdom

**Denise Gigante**

Brazil

**Ian Givens**

United Kingdom

**Colleen Gobert**

Canada

**Robert Goldberg**

United States of America

**Adele Green**

Australia

**Frank Greenway**

United States of America

**Frank Greer**

United States of America

**Ettore Griffo**

Italy

**Carley Grimes**

Australia

**Jolanta Gromadzinska**

Poland

**Giuseppe Grosso**

Italy

**Pilar Guallar-Castillón**

Spain

**Craig Gundersen**

United States of America

**Vanessa Ha**

Canada

**Peter Hannan**

United States of America

**Joanna Harasym**

Poland

**Abdelmonem Hassan**

Qatar

**Wei He**

United States of America

**Anne-Louise Heath**

New Zealand

**Jean-Luc Heeb**

Switzerland

**Kevin Heffernan**

United States of America

**Venkatesh Hegde**

United States of America

**Nazisa Hejazi**

Malaysia

**Helen Hermana Hermsdorff**

Brazil

**Teri L. Hernandez**

United States of America

**Alyson Hill**

United Kingdom

**Masao Hirayama**

Japan

**Tadakazu Hisamatsu**

Japan

**Jonathan Hodgson**

Australia

**Justus Hofmeyr**

South Africa

**Trygve Holmoy**

Norway

**Astrid Horstman**

Netherlands

**Michael Hourdakis**

Greece

**Melanie Howes**

United Kingdom

**Guowei Huang**

China

**Xianju Huang**

China

**Fatma Huffman**

United States of America

**Tommaso Iannitti**

United Kingdom

**Sabine Ibrügger**

Denmark

**Fumiaki Imamura**

United Kingdom

**Concetta Irace**

Italy

**Fatima Isa**

United Kingdom

**Mamoru Isemura**

Japan

**Masanori Iwase**

Japan

**Sandra Jacobson**

United States of America

**Tina Jafari**

Iran

**Greta Jakobsdottir**

Sweden

**Anthony Paul James**

Australia

**Wasantha Jayawardene**

United States of America

**Maria Elena Jefferds**

United States of America

**You-Jin Jeon**

Korea, South

**Inken Jobse**

Germany

**Elizabeth Johnson**

United States of America

**Carol Johnston**

United States of America

**Margalida Joy**

Spain

**Jordi Julvez**

Spain

**Andriana Kaliora**

Greece

**Paul Kalliokoski**

Sweden

**Masao Kaneki**

United States of America

**Paige Kaplan**

United States of America

**Maria Kapsokefalou**

Greece

**Teruo Kawada**

Japan

**Sarah Kehoe**

United Kingdom

**Chad Kerksick**

United States of America

**Amina Khambalia**

Australia

**Olga Khavjou**

United States of America

**Geok Lin Khor**

Malaysia

**Jong Yeol Kim**

Korea, South

**Stanislaw Klek**

Poland

**Kazuki Kobayashi**

Japan

**Mallory Koenings**

United States of America

**John Koethe**

United States of America

**Rene Koopman**

Australia

**Bart Koot**

Netherlands

**Arkadiusz Kozubek**

Poland

**Richard Kreider**

United States of America

**Hanne Kronborg**

Denmark

**Anna Kucharska-Newton**

United States of America

**Remko Kuipers**

Netherlands

**Hiromichi Kumagai**

Japan

**Teru Kumagi**

Japan

**Benoit Lamarche**

Canada

**Linda Landini**

Italy

**Lies Langouche**

Belgium

**John Lasekan**

United States of America

**Martine Laville**

France

**Chrystalleni Lazarou**

Greece

**Marcelo Leal**

Brazil

**Naruemon Leelayuwat**

Thailand

**Angela Leung**

United States of America

**Shengxu Li**

United States of America

**Li-Chan Lin**

Taiwan

**YK Lin**

Taiwan

**Bengt Lindholm**

Sweden

**David Lineback**

United States of America

**Jonathan Little**

Canada

**Keith Lividini**

United States of America

**Adrian Lopresti**

Australia

**Alice Lucey**

Ireland

**Claudia Luevano-Contreras**

United States of America

**Natalie Luscombe**

Australia

**Irja Lutsar**

Estonia

**Ruth Macdonald**

United States of America

**Lesley Macdonald-Wicks**

Australia

**Amanda Macfarlane**

Canada

**Subbarao Madhunapantula**

India

**Melinda Maggard**

United States of America

**Chandana Maitra**

Australia

**Kevin Maki**

United States of America

**Grace Marquis**

Canada

**Bernadette Marriott**

United States of America

**Jennifer Martin-Biggers**

United States of America

**Elisabet Mauri Obradors**

Spain

**Elba Mauriz**

Spain

**Mark Mccarty**

United States of America

**Megan Mccrory**

United States of America

**Robert Mccue**

United States of America

**Cameron Mcdonald**

Australia

**Lynne Mcfarland**

United States of America

**Gael Mearns**

New Zealand

**Andreas Michalsen**

Germany

**Walter A Mihatsch**

Germany

**Ivon Milder**

Netherlands

**Irina Milisav**

Slovenia

**Marcos Minicucci**

Brazil

**Anoop Misra**

India

**Thaís Moreira**

Brazil

**Umberto Morelli**

France

**Hidekazu Moriya**

Japan

**Jean-Claude Moubarac**

Brazil

**Jane Muir**

Australia

**Kenneth Mukamal**

United States of America

**Matthew Muldoon**

United States of America

**Ian Murray**

United Kingdom

**Kazufumi Nakamura**

Japan

**Yoshio Nakata**

Japan

**Jolly Nankunda**

Uganda

**Flavio Nervi**

Chile

**Claudia Netto**

Brazil

**David C. Nieman**

United States of America

**Priyanka Nigam**

India

**Katharina Nimptsch**

United States of America

**Gavin Norton**

South Africa

**Paulina Nowicka**

Sweden

**Bennett Nwanguma**

Nigeria

**Sarah Nyante**

United States of America

**Caio Oliveira**

Brazil

**Jot Hui Ooi**

United States of America

**Maretha Opperman**

South Africa

**Erin O'Reilly**

Canada

**Michael Ormsbee**

United States of America

**Erika Ota**

Japan

**Rei Otsuka**

Japan

**Hsiao-Liang Pai**

United States of America

**Lachlan Palmer**

Australia

**Antonio Paoli**

Italy

**Isabelle Papet**

France

**Eunyoung Park**

United States of America

**Sohyun Park**

United States of America

**Natalie Parletta**

Australia

**Timo Partonen**

Finland

**Amanda Patterson**

Australia

**Elizabeth João Pavin**

Brazil

**Nathércia Percegoni**

Brazil

**Rosangela Pereira**

Brazil

**Miroslav Petr**

Czech Republic

**Regina Phillips**

United States of America

**Olivia Phung**

United States of America

**Grant Pierce**

Canada

**Nuno Pimenta**

Portugal

**Evangelos Polychronopoulos**

Greece

**Shideh Pouria**

United Kingdom

**Joe Proietto**

Australia

**Bo Qin**

United States of America

**Li-Qiang Qin**

China

**Susan Raatz**

United States of America

**Susan Racette**

United States of America

**Retnagowri Rajandram**

Malaysia

**Rita Raman**

United States of America

**Dan Ramdath**

Canada

**Heather Rasmussen**

United States of America

**Norman Ratcliffe**

United Kingdom

**Hollie Raynor**

United States of America

**Asnat Raziel**

Israel

**Anne Rentfro**

United States of America

**Marcelo Ribeiro**

Brazil

**Nico Rizzo**

United States of America

**Lindsay Robinson**

Canada

**Rosalia Rodriguez-Rodriguez**

Spain

**Joshua Roffman**

United States of America

**Nipa Rojroongwasinkul**

Thailand

**Martin Root**

United States of America

**Filippo Rossi**

Italy

**Anne-Françoise Rousseau**

Belgium

**Kevin Rowley**

Australia

**Frank Sacks**

United States of America

**Gulhan Samur**

Turkey

**Francisco J Sánchez-Muniz**

Spain

**Shridhar Sathe**

United States of America

**Giovanni Scapagnini**

Italy

**Jeffery Schau**

Canada

**Susan Schembre**

United States of America

**Barbara Schneeman**

United States of America

**Stéphane Schneider**

France

**Gina Segovia-Siapco**

United States of America

**R. Andew Shanely**

United States of America

**Nick Shaw**

Australia

**Sangeetha Shyam**

Malaysia

**Vishal Singh**

India

**Sheila Skeaff**

New Zealand

**Helen Skouteris**

Australia

**Ann Skulas-Ray**

United States of America

**Inez Slamet-Loedin**

Philippines

**Joanne Slavin**

United States of America

**David Smith**

United Kingdom

**David Smith**

United Kingdom

**Kylie Smith**

Australia

**Noel W. Solonons**

Guatemala

**Elysée Somasse**

Belgium

**Marijn Speeckaert**

Belgium

**Sally Stabler**

United States of America

**Kimon Stamatelopoulos**

Greece

**Zeno Stanga**

Switzerland

**Catherine Stanton**

Ireland

**Blanka Stiburkova**

Czech Republic

**Manfred Stommel**

United States of America

**Javier Stosic**

Argentina

**Jeff Stout**

United States of America

**Saverio Stranges**

United Kingdom

**Nis Stride**

Denmark

**Jean-Marc Tadie**

France

**Markus Takkunen**

Finland

**Kiyoshi Tanaka**

Japan

**Linda Tapsell**

Australia

**Paul Terry**

United States of America

**Anastasios Theodorou**

Cyprus

**Frank Thies**

United Kingdom

**Tinku Thomas**

India

**George Thomson**

United Kingdom

**Gianluca Tognon**

Sweden

**Hirofumi Tomiyama**

Japan

**Duong Tran**

United States of America

**Chin-Hsiao Tseng**

Taiwan

**Koichi Tsuneyama**

Japan

**Tao-Hsin Tung**

Taiwan

**Giovanna Turconi**

Italy

**Jairam Vanamala**

United States of America

**Krista Varady**

United States of America

**Juliana Vaz**

Brazil

**Alfonso Vidal Casariego**

Spain

**Matam Vijay-Kumar**

United States of America

**Ramon Vilallonga**

Spain

**Heather Vincent**

United States of America

**Nishant Visavadiya**

United States of America

**Stella Volpe**

United States of America

**Lidia Wadolowska**

Poland

**Dan Waitzberg**

Brazil

**Yujie Wang**

United States of America

**Xuewen Wang**

United States of America

**Mary Ward**

United Kingdom

**Tamar Weenen**

Netherlands

**Martin O. Weickert**

United Kingdom

**Friede Wenhold**

South Africa

**Jay Whelan**

United States of America

**Stephney Whillier**

Australia

**Anne Wijtzes**

Netherlands

**Jacob Wilson**

United States of America

**Semon Wu**

Taiwan

**Andrzej Wykretowicz**

Poland

**Wenzhong Xiao**

United States of America

**Wanghong Xu**

China

**Weili Xu**

Sweden

**Sho-Ichi Yamagishi**

Japan

**Yong Yang**

United States of America

**Beng San Yeoh**

United States of America

**Yusuf Yilmaz**

Turkey

**Ian Young**

United Kingdom

**Danxia Yu**

United States of America

**Keun-Sang Yum**

Korea, South

**Raul Zamora-Ros**

Spain

**Baoshun Zhang**

China

**Jian Zhang**

China

**Kun Zhu**

Australia

**Zongjian Zhu**

United States of America

**Angela Zivkovic**

United States of America

**Claire Zizza**

United States of America

